# The importance of histopathology in the diagnosis of isolated renal sarcoidosis: a case report

**DOI:** 10.1590/2175-8239-JBN-2018-0069

**Published:** 2018-06-18

**Authors:** João Onofre Trindade, Kaline Daniele de Souza Amaro, Allana Desirée Teixeira de Oliveira, Cecília Neta Alves Pegado Gomes, Hermann Ferreira Costa, Vinicius Nogueira Trajano

**Affiliations:** 1 Faculdade de Medicina Nova Esperança, João Pessoa, PB, Brasil.

**Keywords:** Sarcoidosis, Nephrology, Histology, Sarcoidose, Nefrologia, Histologia

## Abstract

**Introduction:**

Sarcoidosis is a systemic inflammatory disease of unknown etiology, characterized by the presence of non-caseating granulomas in several organs; renal impairment alone is a rare condition. When it affects the kidneys, the most prevalent manifestations are hypercalcemia and hypercalciuria. This paper aims to address the topic of renal sarcoidosis, by means of a case report, and reinstate the importance of histopathology in its diagnosis.

**Methods:**

The data came from an observational clinical study with a qualitative approach, through an interview with the renal sarcoidosis patient and data from her medical records.

**Case report:**

Patient D.M.S., 50 years old, Caucasian, presented with reddish eyes and body pains lasting for fifteen days as first manifestations of the disease. Upon kidney ultrasound scan, we found renal parenchymal nephropathy. Serial renal function and metabolic tests reported anemia and progressive urea and creatinine changes, as well as hypercalcemia and hypercalciuria, confirming acute kidney failure (AKF). A histopathological examination suggested the diagnosis, which was confirmed by clinical, laboratory and histopathological data. There was therapeutic resolution after steroid therapy.

**Discussion:**

The symptomatology of sarcoidosis is diverse and often non-specific. Renal manifestation, which usually occurs after organ involvement, is present in less than 5% of patients, and about 1% to 2% of these patients may develop AKF.

**Conclusions:**

The use of histopathology together with clinical and laboratory data to diagnose isolated renal sarcoidosis, rule out other etiologies and introduce early treatment is of paramount importance.

## RESUMO

**Introdução:**

A sarcoidose é uma doença inflamatória sistêmica de etiologia desconhecida caracterizada pela presença de granulomas não caseosos em diversos órgãos, sendo raro o comprometimento puramente renal. Quando acomete os rins, as manifestações mais prevalentes são hipercalcemia e hipercalciúria. Este trabalho objetiva abordar o tema sarcoidose renal, por meio de relato de caso, e reafirmar a importância da histopatologia no diagnóstico.

**Métodos:**

Os dados foram obtidos por estudo clínico observacional com abordagem qualitativa, por meio de entrevista com a paciente portadora de sarcoidose renal e dados de seu prontuário médico.

**Relato de caso:**

Paciente D.M.S., 50 anos, caucasiana, apresentou como primeiras manifestações da doença olhos avermelhados e dores no corpo com duração de quinze dias. Em ultrassonografia renal, foi constatada nefropatia parenquimatosa renal bilateral. Testes seriados de função e metabolismo renal relataram anemia e alteração progressiva de ureia e creatinina, além de hipercalcemia e hipercalciúria, constatando quadro de insuficiência renal aguda (IRA). Foi indicado exame histopatológico que sugeriu o diagnóstico, confirmado pelos dados clínicos, laboratoriais e histopatológico somados. Houve resolução terapêutica após corticoterapia.

**Discussão:**

A sintomatologia da sarcoidose é diversificada e, muitas vezes, inespecífica. A manifestação renal, que ocorre geralmente após o acometimento de outros órgãos, está presente em menos de 5% dos pacientes, e cerca de 1% a 2% destes podem desenvolver IRA.

**Conclusões:**

É de suma importância o auxílio da histopatologia somada aos dados clínicos e laboratoriais para diagnóstico de sarcoidose renal isolada, exclusão de outras etiologias e introdução de terapêutica precoce.

## INTRODUCTION

Sarcoidosis is a chronic multisystem inflammatory disease, characterized by epithelial granulomas and non-necrotizing giant cell granulomas. Among the affected organs, the lungs are the most prevalent, and kidney involvement is rare. When it occurs, disorders of bone and mineral metabolism such as hypercalcemia and hypercalciuria, which predispose to prerenal azotemia, acute tubular necrosis, nephrolithiasis and nephrocalcinosis, are common.^1^ The concomitance of pulmonary lesions is reported in 90% of the renal impairment cases. However, renal sarcoidosis is rarely confirmed in life.^2^ The incidence is higher in developed countries, slightly affecting women more than men, with peak incidence in young adults, between 25 and 29 years of age, and a second peak in persons between the ages of 65 and 69.^3^

Regarding etiology, the cause is unknown, but the most accepted hypothesis is that of a genetically susceptible host and environmental exposure. Some studies suggest an increased risk of HLA allele-related sarcoidosis.

Early diagnosis and appropriate treatment preserve renal function and prevent progression to the chronic form, which can trigger chronic kidney disease and even irreversible renal failure.

The present study aims to report a clinical case of a rare overall involvement, isolated renal sarcoidosis, and reaffirms the importance of the early histopathological diagnosis to confirm or rule out the etiological suspicion and provide for adequate therapeutics.

## METHODOLOGY

After approval by the Ethics and Research Committee (CEP) of the Faculdade de Medicina Nova Esperança, a descriptive study of the Clinical Case Report was carried out, with a quantitative and qualitative approach, on isolated renal sarcoidosis, through an interview with the patient, after signing the Informed Consent Term (TCLE), data was collected from her medical records into a pre-established form, with subsequent scientific discussion of the case under analysis.

## CASE REPORT

D.M.S., female patient, 50 years old, Caucasian, single, pharmacist, initially went to a hospital emergency room complaining of red eyes and body aches in the last fifteen days as the first manifestations of the disease, which was treated as conjunctivitis but without resolution. She had had a history of renal microcapsules for two years, and bilateral renal parenchymal nephropathy, due to increased medullary echogenicity found on kidney ultrasonography. Therefore, serial renal function and metabolic tests were ordered; which reported anemia, hematocrit drop from 25.5% to 24.9% (RV: 36-45), and progressive urea elevation of 75 mg/dL to 132 mg/dL (RV: 16-40); and creatinine from 1.2 mg/dL to 2.5 mg/dL (RV: 0.6-1.2) upon seven days of follow-up, confirming an Acute Kidney Injury (AKI).

We started her on prednisone at 2 mg/kg daily for 3 months, with reduction of 5 mg per week after that period, until complete suspension. In the meantime, serology for hepatitis B and C, anti-DNA, C3 and C4, anti-streptolysin O antibody (ASLO) and gamma globulin, all with negative results, besides normal albumin of 4.4 g/dL (RV: 3,5-4,8); ruling out several possible infectious and autoimmune etiologies. However, there was a persistent increase in C-Reactive Protein (CRP) of 11 mg/L (RV: < 8), which is an inflammatory marker; increasing proteinuria reaching 856.3 mg/24h in 60 days of follow-up (RV: up to 150), hypercalcemia of 13.1 mg/dL (RV: 8.5-10.2) and hypercalciuria of 504.7 mg/24h (VR: 100-300) (Table 1).

**Table 1:** Follow-up of laboratory data

	Admission	7 days	30 days	2 months	6 months	9 months
Creatinine (mg/dL)	1.5	2.5	3.04		1.1	
Urea (mg/dL)	75	132				
Cr Clearance* (mL/min/1.73m^2^)			69.3			29.11
Calcemia (mg/dL)		8.9		13.1	10.2	12.7

* Cr Clearance: creatinine clearance.

Therefore, due to the acute renal damage associated with hypercalcemia and hypercalciuria, the patient had an indication of renal biopsy. Histopathological examination revealed inflammatory granulomatous reactions in the renal interstitium, a finding that is not uncommon in cases of ruptured tubular lesion and exposure of the content into the interstitium, suggesting a diagnosis of Renal Sarcoidosis. However, adding such a finding to the clinical and laboratory data of the patient, by ruling out other causes, and after evaluation by ophthalmologist and pneumologist - reporting absence of other manifestations, a diagnosis of Isolated Renal Sarcoidosis was confirmed.

High-dose corticosteroid therapy was completed and successful, and the patient reported improved clinical status and laboratory stability.

## DISCUSSION

The symptoms of sarcoidosis are very broad, ranging from asymptomatic individuals or those with non-specific symptoms, such as fever, weight loss, night sweats and fatigue; to pulmonary, ocular, cutaneous, musculoskeletal, and lymphadenopathy involvement, depending on the affected organ. Neurological and cardiac symptoms are rare, as are renal manifestations,^3^^,^ 4 occurring in less than 5% of patients, of which approximately 1% to 2% may develop acute kidney failure (AKI).^3^ The present case revealed only renal manifestations, confirmed after evaluation by other specialists, and that progressed to AKF, evidenced by the expressive increase of urea and creatinine, in only seven days of follow-up, within the select world group of patients with renal sarcoidosis only.

Kidney manifestations usually appear after diagnosis in other organs, or concomitantly.^5^ The most common forms of presentation of renal sarcoidosis are related to calcium metabolism alterations, especially hypercalcemia, hypercalciuria, obstructive uropathy, tubulointerstitial diseases and glomerular diseases.^5^ Our patient presented significant hypercalcemia and hypercalciuria, and kidney micro-stones, which had been reported for 2 years in the patient's history and may have been due to sarcoidosis, since hypercalciuria is the major cause of acute changes in kidney function and defects in urinary concentration , nephrocalcinosis, renal tubular acidosis and renal lithiasis, making it difficult to diagnose.^6^

Prolonged hypercalcemia that develops in sarcoidosis induces intrarenal vasoconstriction, which with disease evolution and delayed diagnosis and treatment, leads to persistent ischemia and may cause decreased glomerular filtration and ischemic acute tubular necrosis. Hypercalcemia is a common finding in sarcoidosis. It is found in 10% to 20% of patients and it is due to increased intestinal calcium absorption and bone resorption secondary to elevated levels of active vitamin D (calcitriol). The activated macrophages present in granulomas are responsible for the calcitriol increase.^6^

Although the anemia has a multifactorial origin, this patient's anemia may have originated from her kidney disease, since the progression of the disease is accompanied by a decrease in erythropoietin - which causes hypoproliferation of red blood cells, leading to normocytic and normochromic anemia. Anemia usually develops when creatinine clearance is lower than 35-45 ml/min.^8^ In addition, angiotensin-converting enzyme is produced by granulomas, and its dosage may be elevated in 60% of the cases.^7^

Therefore, the gold standard for the diagnosis of sarcoidosis is histopathology by renal biopsy, especially in the absence of other extrarenal signs. It is valuable in determining diagnosis and prognosis, and in the planning of short-term treatment for acute intrinsic renal failure, as well as the renal sarcoidosis itself and its metabolic complications. The most frequent parenchymal renal involvement (79%) is NGTI (non-caseating granulomatous tubulointerstitial nephritis), usually an acute impairment of renal function when symptomatic.^8^

**Figure 1:**
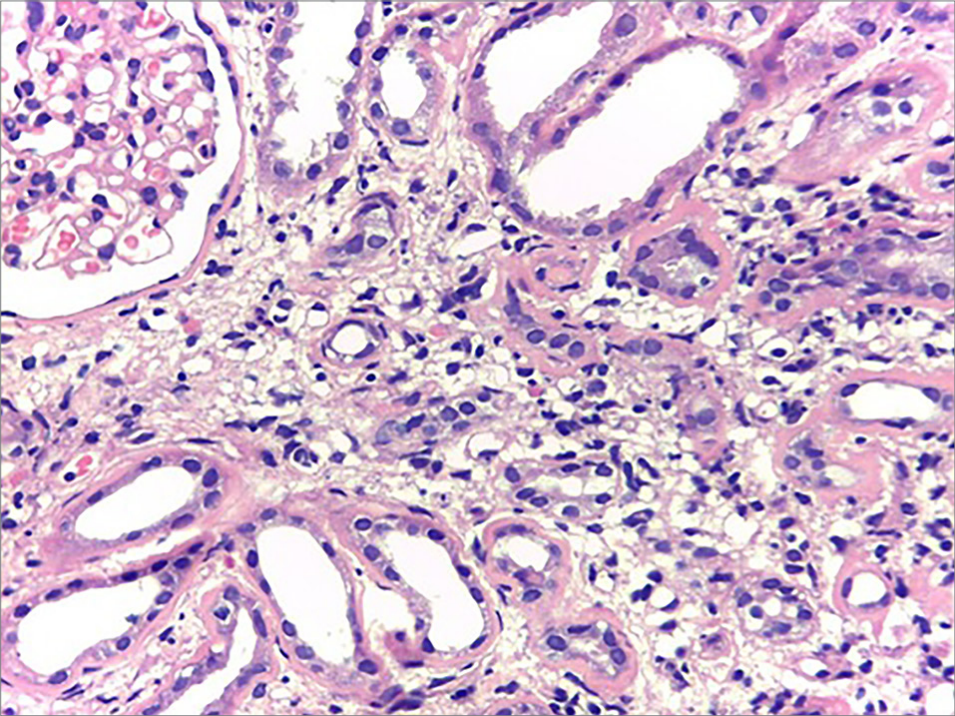
Tubular atrophy with interstitial fibrosis, edema and inflammatory infiltrate of lymphoid cells (H & E - 100x). Collaboration of pathologist Dr. Luiz A. Moura.

**Figure 2:**
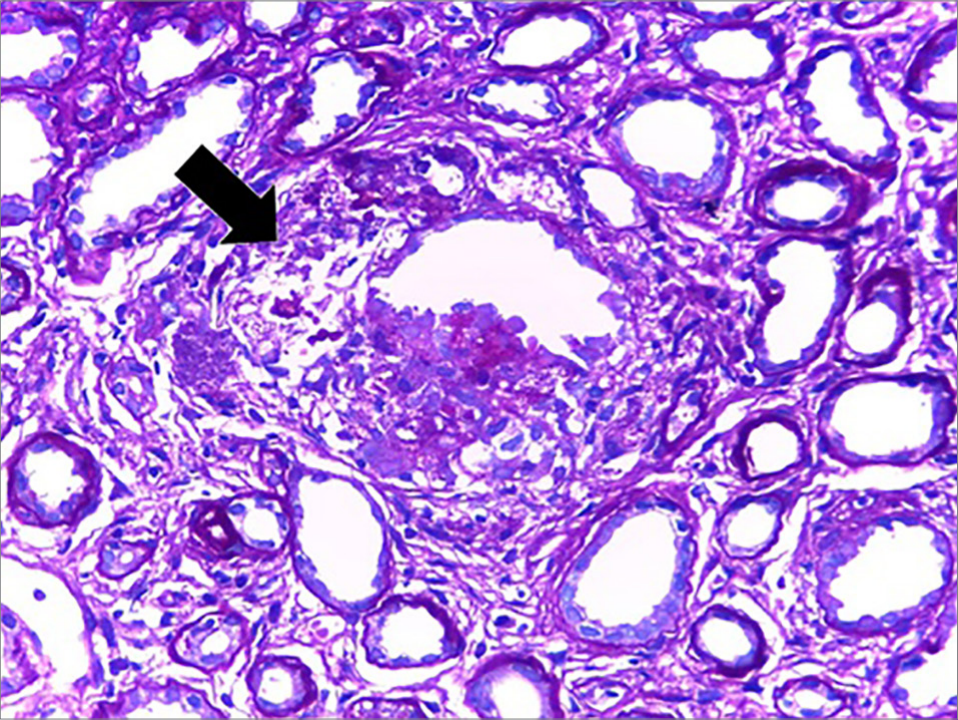
Tubule with altered architecture and surrounded by granulomatous inflammatory reaction of tuberculoid pattern (arrow) (PAS - 100x). Collaboration of pathologist Dr. Luiz Moura.

**Figure 3:**
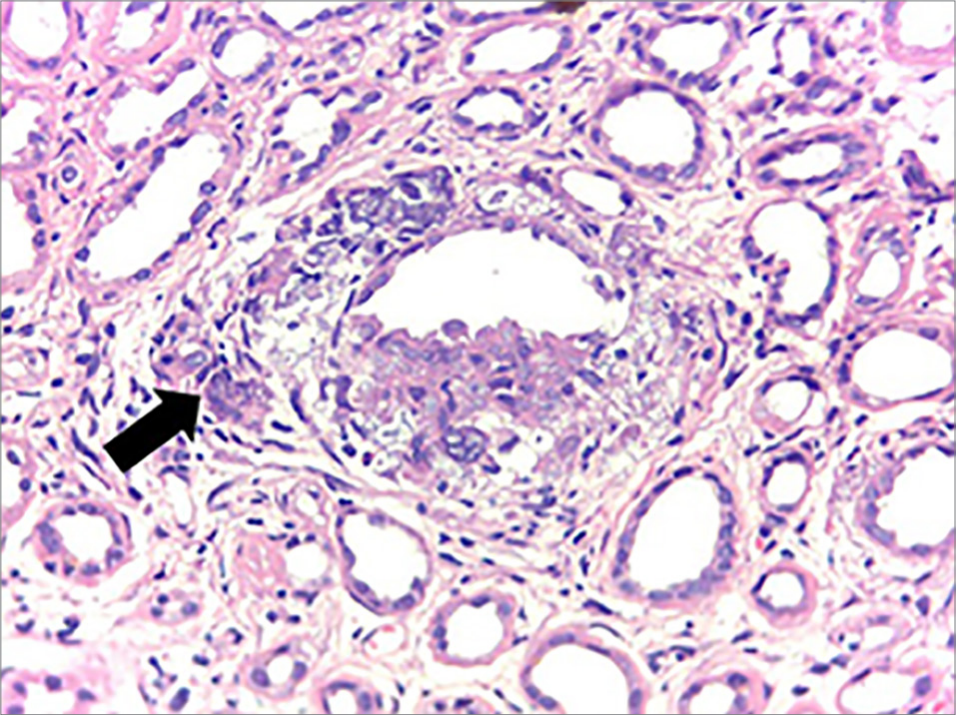
Inflammatory process area in granulomatous pattern, with giant multinucleated cell (arrow), interspersed by foci of dystrophic calcification, arranged around tubular structure (H & E - 100x). Collaboration of pathologist Dr. Luiz Moura.

On the other hand, upon light microscopy, the tissues affected by sarcoidosis develop non-caseiform granulomas, composed of densely arranged epithelioid cells with well-differentiated tubercules,^9^ that is consistent with the histopathological findings in our patient, who also had interstitial foci of histiocytic inflammatory cells in a nodular arrangement around the tubules and interspersed by multinucleated giant cells. The patient also presented multifocal tubular atrophy with moderate interstitial fibrosis, chronic tubulointerstitial nephritis with granulomatous foci of tuberculoid pattern (Figures 1, 2 and 3). However, this finding is not uncommon in cases of other tubular lesions with rupture and exposure of the contents to the interstitium, from drug, infectious and inflammatory causes (Table 2), which made the diagnosis of Renal Sarcoidosis a suggestion based on the histopathological analysis.

**Table 2:** Causes for inflammatory interstitial nephritis

Drugs	Omeprazole, furosemide, allopurinol, captopril, paracetamol, NSAIDs, penicillin, quinolones, acyclovir, vancomycin, rifampin.
Infectious	Tuberculosis, leprosy, toxoplasmosis, candidiasis, cryptococcosis.
Inflammatory	Granulomatosis with polyangiitis, eosinophilic granulomatosis with polyangiitis, sarcoidosis.
Others	Uric acid pigment, heroin, tubulointerstitial nephritis with uveitis (TINU).

The treatment was performed with high doses of corticosteroids, since it improves renal function quickly, but many patients do not recover completely.^10^ Our patient presented a satisfactory response to the therapy instituted, with improvements in her clinical and laboratory conditions. Corticosteroid therapy is the basis of treatment, with 20 to 40 mg of prednisone/day for 6 to 12 weeks, with subsequent dose reduction. If there is therapeutic failure or if there are contraindications, immunosuppressive agents, such as azathioprine and mycophenolate mofetil, or the TNF-alpha infliximab antagonist, have been increasingly used in recent years, with a good response in refractory cases.^11^

## CONCLUSIONS

Renal sarcoidosis alone is rarely diagnosed, since the disease affects a small portion of the population and is difficult to detect, ranging from asymptomatic patients to those with Chronic Kidney Disease. Early diagnosis and appropriate treatment preserve renal function and avoid progression to chronic secondary forms. Therefore, rapid decay of renal function must be investigated by means of renal function tests, as well as biopsy of the organ for histopathological examination, in order to diagnose the clinical presentation presented by the patient and, from this, start early and efficient treatment.

In this case study, the clinical, laboratorial, radiological and histopathological findings together confirmed, by ruling out other causes, the diagnosis of Isolated Renal Sarcoidosis, establishing the expected corticosteroid therapy with complete clinical and laboratory improvement of the condition.
